# A Review on Newer Interventions for the Prevention of Diabetic Foot Disease

**DOI:** 10.7759/cureus.30591

**Published:** 2022-10-22

**Authors:** Sanket Tekale, Anuj Varma, Shubhangi Tekale, Unnati Kumbhare

**Affiliations:** 1 Department of Medicine, Jawaharlal Nehru Medical College, Datta Meghe Institute of Medical Sciences (Deemed to be University), Wardha, IND; 2 Department of Pathology, Dr. Ulhas Patil Medical College and Hospital, Jalgaon, IND

**Keywords:** foot injuries, remote monitoring, sensors, prevention, technologies, telehealth, diabetic foot disease

## Abstract

Diabetic foot disease (DFD), which includes ulcers on the foot, infections, and gangrene of the foot, is one of the leading causes of disability worldwide. About half of diabetic foot disease (DFD) patients have a recurrence in less than a year. To alleviate the burden of DFD globally, it is essential to give long-term medication to reduce the likelihood of recurrence. The effectiveness of telemedicine, wearable technologies, and sensors in DFD prevention is discussed in this review. Offloading footwear helps to cure and prevent ulcerated diabetic foot by distributing physical stress away from bony prominences. Sensors and wearables can record the temperatures of the foot, blood pressure (BP), and blood sugar levels and estimate lipid profile. These technologies have offered a practical means of reaching individuals in rural areas with a heightened risk of developing DFD. There is less need for in-person consultations with this strategy. This methodology is simple to operate and lessens reliance on patients. The benefits of adopting these remote monitoring approaches have been demonstrated in some studies with DFD-at-risk individuals. It is required to do more analysis to ascertain the effectiveness and value of incorporating different remote monitoring systems as part of an all-encompassing strategy to prevent DFD.

## Introduction and background

Diabetic foot disease (DFD) is a major global challenge for the geriatric age group [1]. Almost 18%-34% of diabetic patients will develop diabetic foot disease in their life [2]. Life expectancy is affected due to complications such as infections and limb amputation [2]. DFD is the leading cause of disabilities worldwide, including ulcers on the foot, infections, and gangrene [3]. DFD is a chronic disease since 40% of those with it experience a recurrence within a year. Therefore, research into this condition should concentrate on preventing remission and serious effects, including amputation and death [2]. Although diabetic foot ulcer (DFU) can arise from acute trauma, it often results through recurrent episodes of physical exertion brought on by weight-bearing activity that is undetectable due to the development of diabetic peripheral neuropathy (DPN) [4]. Consequently, wearing the proper footwear to "offload" high-stress areas on the bottom of the foot is essential in managing DFD and avoiding the development of new ulcers [5,6].

The leading causes for DFD recurrence and the escalation of complications include higher pressure on plantar surfaces, an unsteady gait, hyperglycemia, hypertension (HTN), high cholesterol, and triglycerides levels [7-9]. Results from various randomized controlled trials and analyses suggest that these primary reversible risk factors can be reduced using treatment modalities such as good foot hygiene, appropriate footwear usage, and medical management [7-10]. There are several sensors and wearables on the market or being developed right now for monitoring diabetic individuals in remote areas of these critical risk variables. This gives a technique to care for patients at risk of DFD when linked with telemedicine management. Implementing these techniques may decrease the possibility of patients and staff developing respiratory illnesses and infectious infections [11,12].

## Review

Risk factors of diabetic foot ulcer

The risk factors for the progression of diabetes-related foot ulcers [13] and how the sensors and wearables could help in the remote monitoring of these factors have been depicted in Table 1.

**Table 1:** Examples of techniques with potential value of preventing diabetic foot disease. [13-19]

Risk factors	Current plan of treatment	Interventions developed	Impact of intervention
Foot ulcers	Podiatrist consultation	Foot temperature monitoring smartphone apps	Reduced recurrence of foot ulcers at high-risk areas
Increased pressure on the foot	Offloading devices	Foot pressure monitoring	Increased use of offloading devices
Increased tissue stress	Patient counseling	Footwear adherence monitor	Increased contact time of offloading devices
Increased blood glucose level	Anti-glycemic medications	Continuous glucose monitor	Reduced vascular complications
Hypertension (HTN)	Anti-hypertensive therapy	Cuffless blood pressure recorder	Reduced cardiac and vascular complications
Abnormal canter	Surgery	Gait and activity monitor	Improvement in gait and ulcer recurrence
Peripheral vasculopathy	Laboratory investigations and Doppler	Foot blood supply sensor	Reduced vascular abnormalities

Interventions for the prevention of diabetic foot disease

**Figure 1:**
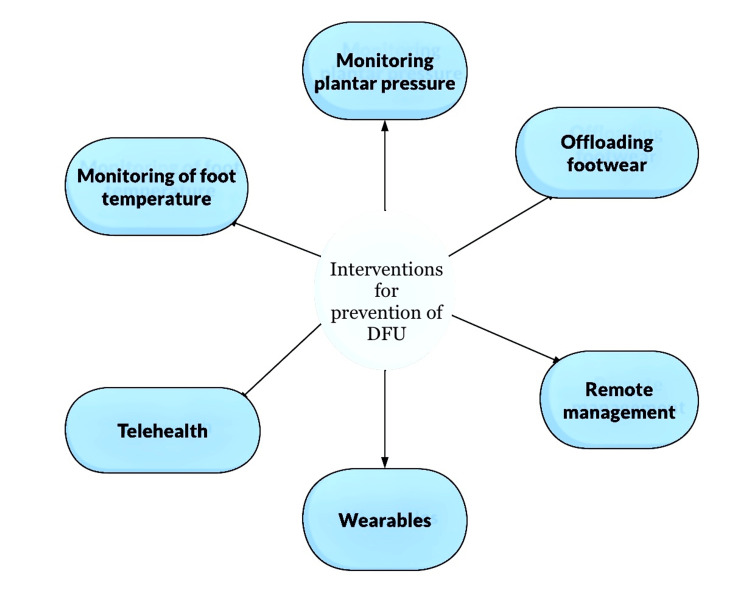
Interventions for the prevention of diabetic foot disease. DFU: diabetic foot ulcer Image credit: the authors of the current review

There are various techniques that can prevent a diabetic patient from developing a foot ulcer. Some of the techniques are being depicted in Figure 1.

Monitoring of Foot Temperature

Individuals with a decrease in protective awareness, such as those suffering from diabetic peripheral neuropathy (DPN), are more prone to develop ulcers on the foot due to repeated damage to the feet [2]. Recurrent trauma causes a "hot spot," or localized inflammation, which may be identified by the increased temperature at the afflicted place [20]. This provides a way to identify those susceptible to developing diabetic foot ulcers (DFU) for the early care of the foot, such as the expulsion of calluses and alterations to footwear to enable better offloading [2]. Infrared thermometers have been utilized in most investigations to measure the foot's temperature at several locations on both sides and compare those locations on opposing feet [21-24]. According to earlier studies, a temperature differential of more than 2.2°C between identical spots on separate feet for two consecutive days helps predict the development of ulcers [25,26]. A recent study found that the hallux; first, third, and fifth metatarsal heads; the mid-foot, and the heel all had different median temperatures from ambient temperature and that this difference can predict the development of foot ulcers with great sensitivity but poor specificity [27]. This could make it possible to keep an eye out for hot areas that indicate upcoming ulcers in those who already have unilateral foot ulcers and those who have had significant solitary amputations in the past.

Regular foot temperature monitoring enables you to initiate quick unloading and care of the feet, such as callus treatment, to stop an emerging foot ulcer because of its predictive value. Four randomized controlled trials [21-24] have examined the efficacy of monitoring the foot's temperature at home to notify people vulnerable to developing DFU that incidence was considerably and significantly reduced in those assigned to foot temperature monitoring at home in three of these trials [21-23], done by the same crew and involving a count of 427 individuals. In the other trial [24], which was carried out by a different study team and only involved 41 participants, it was discovered that quick offloading and temperature monitoring of the foot at home had no discernible effect on the development of foot ulcers [24]. The cost-effectiveness and cost-utility of foot temperature monitoring at home are presently being investigated in a giant randomized experiment with 304 participants [13]. In a recent clinical experiment, foot temperature monitoring at an outpatient clinic rather than at home was found to be effective when done just monthly [28]. To provide measures, such as lowering physical activity and improving the unloading of the troubled region, a thermal camera was used to locate "hot patches" [28]. The experiment had 110 individuals with a history of foot ulcers caused by diabetes, and it found no advantage of the interventions in terms of avoiding ulcers on the foot or enhancing the quality of life in terms of health [28]. These contradictory results could be explained by the less frequent foot temperature monitoring, which might have prevented the early detection of individuals in danger. These results point to the potential advantage of using contemporary technology to remotely monitor participants' foot temperature regularly.

The International Working Group on the Diabetic Foot (IWGDF) has issued a cautious recommendation concerning the use of temperature monitoring of the foot at home due to the poor quality of the data [25]. This is likely because temperature monitoring at home and control of the foot's temperature presents some practical difficulties, smaller sample numbers in previous research, and errors in those studies' design. The generalizability of the earlier studies evaluating home foot temperature monitoring [17-19] was limited since they did not include participants who had peripheral artery disease (PAD), a known risk factor for developing foot ulcers [20-22]. Foot temperature has been correlated to both PAD and DPN [23]. In recent thermal imaging research, individuals with PAD had considerably higher foot temperatures than those without PAD [26,27]. In contrast, a previous study revealed a positive association between ankle-brachial pressure index and foot temperature, suggesting that persons with PAD have lower foot temperatures [28]. Whether or not persons with severe PAD are examined may be related to this discrepancy. Additionally, recent results on infrared thermography and previous clinical experience also demonstrate that the rise in foot temperature that occurs right away after successful revascularization is correlated with rising in the ankle-brachial pressure index [29]. The function of temperature monitoring of the foot in individuals with PAD is still unknown, given the proven consequence of limb perfusion insufficiency on the temperature of the feet and the omission of individuals with this issue from past trials [17-20,24]. To better understand the function of temperature measurements at home in individuals suffering from milder PAD, the enduring trials of temperature monitoring at home described above only exempt individuals with severe limb ischemia, which is defined as systolic ankle blood pressure (BP) of 50 mmHg, systolic toe blood pressure of 30 mmHg, or transcutaneous oxygen pressure of 30 mmHg [9].

The Podimetrics Mat (Podimetrics, Inc. Somerville, MA) is the most precise device for measuring the foot's temperature, which has been reported to date [23,30]. With the least amount of patient engagement possible, this wireless mat is intended to track the foot's plantar surface temperature [30]. The mat automatically captures a thermogram of both feet after being trodden on for roughly 20 seconds. The thermogram securely communicates the information to an authorized server run by the manufacturer while correctly measuring temperatures within the range of 15°C-40°C. Based on the thermogram, foot temperature discrepancy is determined analytically. A thermal gradient of 2.2°C between common areas on both feet successfully foreseen 97% of foot ulcers in previous research of 129 persons with a history of diabetes-related pressure ulcers, with a mean lead time of 37 days and a false positive rate of 57% [30]. With almost the same lead time of 35 days, raising the temperature threshold to 3.2°C lowered sensitivity to 70% while lowering the false-positive rate to 32%. At least three days a week, 86% of the individuals used the system. However, beyond the United States of America, this product is not commercially accessible and can be too costly for private usage.

Monitoring Plantar Pressure

Diabetic peripheral neuropathy (DPN) results in changes in foot contour and the loss of foot muscles [2]. These transformations may increase pressure sites on the plantar area of the foot when standing or walking [21]. According to a prior meta-analysis, individuals suffering from DPN who have a history of ulcers on the foot walk with elevated plantar pressures when compared with DPN individuals who never had an ulcer [31]. The IWGDF highly advised that those with a history of foot ulcers should use footwear designed to reduce excessive plantar pressures [25]. Plantar pressures are often estimated in clinical practices using stress plates or insoles with pressure sensors positioned in healthcare facilities [32]. Devices such as the Pedar® (Novel, Munich, Germany) and F-Scan™ (Tekscan Inc., Boston, MA) can accurately detect plantar pressure within footwear [33]. These technologies have been employed to demonstrate the efficacy of therapeutic footwear in lowering plantar pressures [33]. These are being broadened to detect plantar pressures and pressures of other tissues on the plantar surface of the foot as part of regular exercise [34]. However, patient access to these devices is constrained as they are available in a few clinical settings, need unique data-collecting techniques, and cannot be utilized for monitoring at home [35].

These techniques vary in the sensors used for measuring plantar pressure; some have large capacitive sensors, while others use smaller resistance sensors or more temperature-sensitive piezoelectric sensors. Thus, the user's understanding of the benefits and drawbacks of each technique is crucial for the implementation and the quality of the data collected [34-36]. The SurroSense Rx system (Orpyx Medical Technologies, Calgary, Alberta) is one example of a smart insole that helps in monitoring pressures of the plantar surface and notifies wearers immediately [37]. This device consists of an insole with eight sensor systems, three for each metatarsal head, two for the lateral plantar surface, one for the heel, one for the hallux, and one for the smaller toes. When persistent pressure is found, the wearer sees a pressure mapping of each foot showing the area of prolonged stress and hears an alert (pressure greater than 35-50 mmHg and continuing for 15 minutes) [37]. The combination of pressure data over time forms the basis for the alarm thresholds. Within 20 minutes of identification, the region with prolonged pressure has to be offloaded as the proper therapeutic reaction to the warning. In a study of 17 individuals having a previous incidence of ulcers on the foot, those who received a high volume of notifications, at least one alarm every two hours, wore their shoes for a longer time and responded to alerts with higher consistency [37]. Most participants thought the insoles worked well and were beneficial [37].

Offloading Footwear

Plantar peak pressure can be reduced by over 90% compared to barefoot walking with devices for diabetic foot ulcer (DFU) treatment, such as complete contact casts and detachable cast walkers [38]. Offloading shoes that are intended to prevent DFU reduce plantar pressure less than DFU treatment devices since they are smaller and have less effect on gait. Regarding offloading capacity, offloading devices for avoiding DFU have had more room for growth. With the advent of objective plantar pressure measurements over the past 10 years, the craft of creating personalized preventive offloading footwear has improved. There have been two methods that have worked well. One approach is to directly insert plantar pressure readings into the production of insoles using a computerized milling machine [39]. The second way involves having a shoe technician adjust the footwear depending on the pressure values they see while the wearer's feet are wearing usually made footwear [40]. This approach allows for several forefoot pressure measurement cycles and subsequent footwear alterations. The development of offloading footwear using either way of combining plantar pressure data failed to show a decrease in pre-ulcerative lesions when compared to the use of conventional methods. However, both strategies showed a reduction in the recurrence of DFU [39,40]. When focused on patients who had regularly used their offloading footwear, the study that used plantar pressure measurements to guide footwear modifications only suggested a reduction in DFU recurrences [40].

To promote use, there must be reliable, unbiased data on regular footwear usage. Thermistors implanted in therapeutic footwear monitor offloading utilization [40]. Linking such sensors, which can monitor activities, makes it feasible to calculate footwear conformance as a proportion of regular weight-bearing activities [40]. Offloading footwear continuously while engaging in weight-bearing activities is difficult [41,42]. Studies with offloading devices used to treat active wounds provided the initial data about offloading adherence. Total contact castings were formerly the preferred method for unloading DFU among various alternatives [5]. Removable cast walkers, however, have been utilized far more frequently in actual practice [43]. Complete contact casts have repeatedly shown good healing results in clinical studies, even while detachable cast walkers and total contact casts have an equivalent capacity for unloading DFU [6]. Complete contact casts must be cut off to be removed, in contrast to detachable cast walkers that may be easily removed and then put back on at the user's option. Therefore, it has always been considered that the difference in healing results between the two choices is due to the detachable cast walkers' less contact time.

Remote Management

People who acquire DFD typically do not adopt the best glucose, blood pressure, and cholesterol controls [44]. In comparing patients with diabetes who do not have DFD to those who do, those with DFD had a higher risk of all-cause death [45]. The chances of coronary morbidity are approximately 50% over 10 years, and the mortality rate is predicted to be around 6% in patients with a history annually [46]. This underlines how crucial it is to improve medical management in this group. Intense blood glucose control reduces amputations, according to a meta-analysis of prior randomized studies. Glycemic management is crucial for lowering vascular pathologies [47]. Self-monitoring of blood sugar levels is a common source of information for diabetes treatment in clinical settings [47]. For continual glucose monitoring, wearable or implanted sensors are now being developed [47]. These sensors employ enzymatic technology to analyze interstitial fluid rather than blood sugar [48]. For roughly a week, those sensors can detect glucose non-invasively every five minutes; after that, most equipment needs to be replaced [44]. These sensors have been integrated into systems that help persons with type 1 diabetes improve their glycemic control while automating the supply of insulin [49]. Constant monitoring appears to improve glycemic management, according to recent meta-analyses of randomized studies in persons with type 2 diabetes [50]. For patients with poor glycemic control (glycosylated hemoglobin {HbA1c} 9%), using such gadgets is currently advised by North American guidelines [51]. According to a recent experiment, glucose monitoring, which assesses interstitial fluid glucose, could be utilized in primary healthcare settings; however, HbA1c after 12 months indicates that it might not be superior to more conventional approaches [52]. Continuous blood sugar monitoring may provide considerable benefits for people with diabetes susceptible to complications such as DFD. However, because it is costly, only a tiny percentage of patients can now afford it.

A further significant risk factor for problems in DFD patients is high blood pressure. People who are susceptible to developing DFD, those with PAD, have been demonstrated to experience a decrease in the incidence of cardiovascular events while taking anti-hypertensive drugs, including angiotensin-converting enzyme (ACE) inhibitors and angiotensin receptor blockers [53]. However, blood pressure management is typically insufficient in those at risk of DFD [54]. About 40% of the 2773 PAD patients in the latest research had systolic blood pressure higher than the recommended range of 140 mmHg [54]. Currently, an inflatable cuff that is wrapped around the bicep is used to measure pulsation to monitor blood pressure. New wearable devices without cuffs have been designed to assess blood pressure (BP), and they may offer a more convenient approach to continuously monitor blood pressure and enable better control [55,56]. The devices determine BP using various techniques, including pulse transit time, arterial vibrations, and laser Doppler flowmetry. Further research is required to determine the efficacy and worth of these technologies in enhancing the medical care of DFD-at-risk individuals. Lipid management is necessary for those who are at risk of DFD. Aggressive reduction of low-density lipoprotein (LDL) has been shown to positively prevent significant adverse coronary and extremity events [57,58]. Low-density lipoprotein sensors have also been created, but they need to undergo additional research and development before they can be used widely [59].

Medication non-compliance is the intake of less than 80% of recommended medicine dosage [60]. Non-compliance in diabetic treatment is due to some variables, including affordability and regimen complexity [61]. Patients must take their medications as directed to obtain the best possible management of risk factors. Today, sensors may be used to track the use of medications; for instance, Proteus Discover (Proteus Digital Health, Redwood City, CA) gives patients and healthcare professionals information on drug use and physical activity [62]. It consists of a sensor patch, an implanted sensor, a patient smartphone app, and a supplier's web portal. Following ingestion, the sensor turns on and sends out a signal with a predetermined code, which the patch picks up. The ingestible sensor tablet can monitor treatment adherence when used with the drug. The patches may also track step count, heart rate, and activity. The patch's data is transmitted to a patient's mobile device for viewing and then to the cloud and into a website for a practitioner to observe. An app for mobile devices prompts users to take their medications on time. According to a recent study, Proteus Discover may aid in treating HbA1c, LDL, and BP [62]. These detectors could be helpful for individuals susceptible to developing DFD, but further research and interaction with people and other relevant parties are still needed. The different types of sensors for remote medical care described above have not been the subject of clinical testing; more significantly, the control groups used in studies of remote monitoring systems have varied widely. As a result, it is continually necessary to assess how well these devices can be used to enhance medical care for people vulnerable to DFD.

Wearable Technologies for Evaluating the Gait and Peripheral Neuropathy

Infrequent strides are common in DPN patients, which might raise plantar pressures and cause foot ulcers [63]. Gait monitoring is challenging. However, wearables that allow for remote patient monitoring have recently been developed [64]. With this information, a gait rehabilitation program might be created to reduce forefoot pressure and injury risk. The exact function of artificial intelligence (AI) in healthcare is unknown, but devices such as the gait-enhancing mechatronic system have been invented for improving gait and distributing foot pressure. Various physical therapy and rehabilitation approaches may provide additional solutions for improving gait in DPN patients [65].

Automated computer systems have been created for the analysis of DPN, and they may be capable of recognizing people who are susceptible to evolving DFD [66]. A Cochrane review is evaluating the proof for the validity of all suggested simple examinations for DPN screenings to give better comprehensive information [67]. The invention of sensors to measure the blood flow of the foot has also sparked interest [68]. For instance, the vascular early warning system (VEWS) uses infrared light sensors attached to the toe and dorsum of the foot to assess fluctuations in blood volume inside the vessels during the elevation of the foot [68]. Sensors are not examined compared to standard medical care or randomized controlled studies [69]. The fact that treatment differs significantly across health facilities and countries makes it challenging to evaluate these systems in clinical settings. As a result, the role of these system applications in accomplishing healthcare services is still unclear, although it is a promising area for future study. Gait therapy services delivered remotely may reduce the likelihood of DFD.

Telehealth

Counselling of DFD patients is done every week or on a biweekly basis, while smartphone applications for remote monitoring of patients with DFD have been developed widely but not tested or adopted [70]. Regardless of their likely utility in monitoring DFD in remote areas, cell phone photos have been observed to have low diagnostic accuracy and hence shouldn't be used as a primary or single diagnostic tool of DFD [71]. This domain is continually developing, so new mobile applications and monitoring technologies can improve over time.

## Conclusions

Sensors, wearables, and telehealth techniques have been developed to monitor the contribution of diabetic foot disease (DFD) remotely. We believe that the utilization of telemedicine, wearable, and sensor technologies presented in this study and those currently under development will offer a unique method for determining risk factors in DFD patients. How effective they are at preventing DFD needs to be established. The COVID-19 pandemic might function as a provocative factor for the development and widespread testing of innovative technological approaches for staving off DFD to keep the feet healthy and uninjured at home.
